# Effects of caffeic acid phenethyl ester in reducing cerebral edema in rat subjects experiencing brain injury: An in vivo study

**DOI:** 10.1016/j.amsu.2020.08.016

**Published:** 2020-08-15

**Authors:** Rizha Anshori Nasution, Andi Asadul Islam, Mochammad Hatta, Prihantono, Warsinggih, Dany H. Ludong, Ismail, Harakati Wangi, Muh Nassrum Massi, Khairul Ihsan Nasution

**Affiliations:** a Department of Neurosurgery, Pelamonia Hospital, Makassar, Indonesia; b Department of Neurosurgery, Faculty of Medicine, Hasanuddin University, Makassar, Indonesia; c Clinical Microbiologist Program, Faculty of Medicine, Hasanuddin University, Makassar, Indonesia; d Department of Surgery Faculty of Medicine, Hasanuddin University, Makassar, Indonesia; e Doctoral Program of Medical Sciences, Faculty of Medicine, Hasanuddin University, Makassar, Indonesia; f Department of Interna Medicine, Pelamonia Hospital, Makassar, Indonesia; g Departement of Microbiology, Faculty of Medicine, Hasanuddin University, Makassar, Indonesia; h Departement of Neurosurgery, Putri Hijau Hospital, Medan, Indonesia

**Keywords:** Experimental traumatic brain injury, Caffeic acid phenethyl ester, Blood serum, AQP4, Rat model

## Abstract

**Background:**

A head injury is a very dangerous condition that threatens human life. This study examines the use of caffeic acid phenethyl ester (CAPE) in reducing cerebral edema in cases of head injury. The purpose of this study is to demonstrate whether CAPE can improve various parameters related to the expression of Aquaporin-4 (AQP4) mRNA and the serum AQP4 levels in rat subjects.

**Methods:**

This is a randomized controlled study using a posttest-only control group design that uses experimental animals—specifically, male *Rattus norvegicus* (*Sprague Dawley* strain) rats aged 10–12 weeks and weighing 200–300 g. This study used a head injury model according to Marmarou (1994) with minor modifications to the animal model fixation tool. The parameters of the AQP4 mRNA were examined with real-time PCR, while serum AQP4 levels were examined with sandwich ELISA.

**Results:**

The AQP4 mRNA expression in rats that were given CAPE was lower than those not given CAPE, both on the fourth and seventh days; serum AQP4 levels in rats that were given CAPE were also lower than those not given CAPE, both on the fourth and seventh days.

**Conclusion:**

Administration of CAPE in a rat model with head injury can reduce cerebral edema, mediated by AQP4.

## Highlights

• Head injury is a major cause of mechanical brain cell injury and also catalyzes secondary damage.
• Caffeic Acid Phenethyl Ester (CAPE) can reduce inflammatory processes, brain lipid peroxidation, and free radical damage.
• CAPE treatment can cause a decrease in cerebral edema, mediated by Aquaporin-4 (AQP4).

## 1 Introduction

A head injury is a major cause of mechanical brain cell injury, and it can also catalyze a secondary injury that immediately ensues [1]. This secondary non-mechanical injury is progressive, may start from the first hour of the onset, can last for hours or even days [2], and is characterized by cerebral edema [3]. Various parameters are used as a marker for cerebral edema and as a therapeutic target of cerebral edema, including aquaporin-4 (AQP4), which is induced by astrocytes after a head injury [4].

Caffeic Acid Phenethyl Ester (CAPE) is obtained by extracting propolis from honey bees [5]; the CAPE structure contains catechol, which is a strong antioxidant [6]. Propolis has been used for years for its medicinal properties [7], but CAPE has also been shown to reduce inflammatory processes, brain lipid peroxidation [8], and free radical damage [9]. Reports have indicated that this effect of CAPE is due to its effect on xanthine/xanthine oxidase [9,10], nuclear factor-jB (NF-jB), cyclooxygenase 2 (COX-2), 5-lipoxygenase [[11], [12], [13], [14]], inflammatory cytokine production, and cytochrome *c* release from mitochondria [15,16].

Traumatic head injuries trigger many pathological processes that CAPE targets. Therefore, this study hypothesizes that CAPE treatment will cause a decrease in cerebral edema. This study examined whether post-injury CAPE treatment in animals with traumatic brain injury can reduce the effects of traumatic brain injury by decreasing cerebral edema, tissue loss, and neurological dysfunction.

## 2 Materials and methods

### 2.1 Materials

*Sprague Dawley* male rats (200–300 g) were kept in pathogen-free conditions and adapted to laboratory conditions for two weeks; they were given BR-1 feed without any other additional food. The food was regularly given every day, with aqua distillate as their drink. The cage was a standard-shaped cage and was cleaned routinely to maintain hygiene; the rats were given a 12-h dark/12-h light cycle, with controlled temperature and humidity.

CAPE was purchased from Sigma-Aldrich Pte.ltd, Reagan Number 10454-70-9; AQP4 mRNA was obtained from Oligo Macrogen Gasan-dong, Gheumcheon-gu, Seoul; and AQP4 (sandwich ELISA) was obtained from LSBio, catalog No. LS-F4077.

### 2.2 Brain injury administration and brain tissue collection

Ten *Sprague Dawley* rats were brain-injured and then randomly divided into two groups: receiving CAPE, and not receiving CAPE (placebo). The rats were initially anesthetized with ketamine at a dose of 3–10 mg/kg which had been dissolved in aqua distillate. Then, a coronal incision was made across the midline of the brain, and the researchers cautiously burr-holed using a high-speed drill until the dura mater was exposed. Physical impact was created with a modified Marmarou model [17,18], in which a mass weighing 20 g was dropped from a height of 20 cm, passing through a tube as the medium for its transport [19,20]. The portion of the rat's head in which the dura mater had been exposed was placed just below the transport tube, enabling the 20-g mass to land right on the exposed dura and ensuring that the trauma model has caused damage to the brain. One rat underwent pathology examination with hematoxylin staining (the results were bleeding in the brain tissue), while the other rats were treated according to standard craniotomy procedure. The wound was sutured after antibiotic ointment was administered. All surgical procedures were performed aseptically by adhering to the principle of sterility. After the craniotomy and trauma models, all rats were treated at room temperature for recovery and were returned to their cages.

**Fig. 1:**
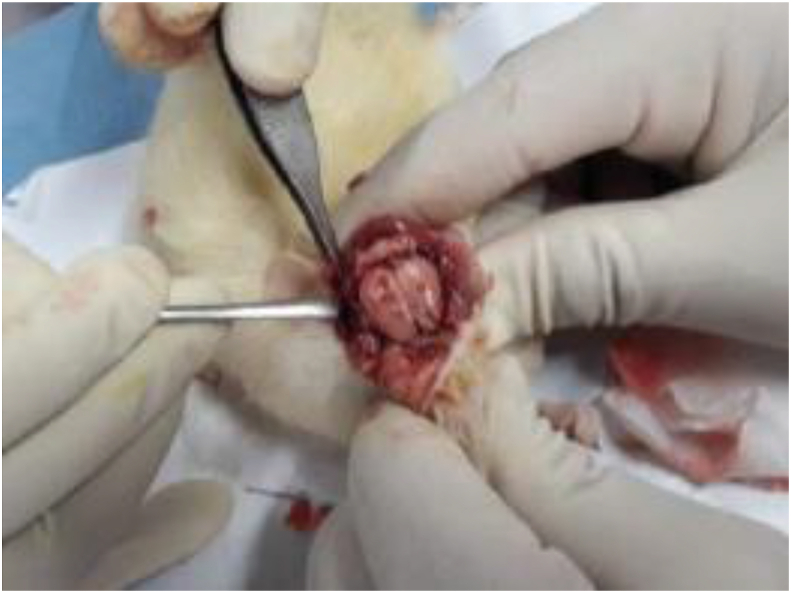
The craniectomy procedure after sacrifice.

On day 7 after the trauma model, the rats were euthanized with 400 mg phenobarbital injection [21], at which point craniectomy was performed to extract brain tissue (Fig. 1). The collected brain tissue were immediately frozen at −80 °C until further processing through histopathologic examination.

This animal experiment was approved by the local ethics commission, number: 771/UN4.6.4.5.31/PP36/2019, and was carried out in line with the ARRIVE Guidelines for Reporting Animal Research [22].

### 2.3 CAPE administration

CAPE was prepared in 50% dimethyl sulfoxide (DMSO) in saline solution and was administered at a dose of 10 mg/kg by intraperitoneal injection (IP) at 30 min post-trauma [23], then re-administered every day for seven days. The control group rats were given a placebo with the same schedule as the CAPE group.

### 2.4 Sample examination

Blood samples were collected 24 h, 4 days, and 7 days after the first CAPE treatment, and brain tissue samples were collected 24 h and 7 days after the first CAPE treatment. Blood samples were examined by AQP4 mRNA expression and sandwich ELISA. Brain tissue processing was conducted through the paraffin and hematoxylin eosin (HE) staining methods in order to reveal the abnormalities.

The mRNA genes were examined using a boom method, with brain tissue samples mixed with an L6 buffer solution then added to diatomic suspense at a vortex and incubated. Quantitative real-time PCR used GAPDH as the “housekeeping gene” (internal control) with the forward primer/sense strand: ACCACAGTCCATGCCATCAC with DNA replication, in a 94 °C cycle for 10 min. The cycle was repeated 32 times at 54 °C (30 s). The reverse primer/anti-sense strand: TCCACCACCCTGTTGCTGTA was used in accordance with the Tomomi Yajima protocol while AQP4 serum levels were measured with sandwich ELISA from LSBio [24].

### 2.5 Statistical analysis

Data were processed and analyzed using SPSS version 23 (IBM Corp, released 2015: IBM SPSS Statistics for Windows, Version 23.0, Armonk, NY: IBM Corp). The effects of CAPE administration—in this study, on the expression of AQP4 mRNA—was tested by independent *t*-test, displayed as mean (±SD [standard deviation]), and a *p*-value of <0.05 was considered significant.

## 3 Results

### 3.1 Characteristics of subjects

This study examined the role of CAPE in inhibiting AQP4 mRNA. By using a head injury model on test rats (*Sprague Dawley*), this study aimed to explain the benefits of CAPE in relation to the process of cerebral edema. The characteristics of the animal subjects, such as body weight, are listed in Table 1.

**Table 1:** *Sprague Dawley* rat body weight.

	Body weight (gram)	p-value
Mean	290.07	0.155
SD	±10.48	

A homogeneity test was conducted of the *Sprague Dawley* rats using the lavene homogeneity test, and a *p*-value of ≤0.05 was obtained; therefore, it can be concluded that there was no significant difference among the body weights of the rats.

### 3.2 Protein expression status in brain-injured *Sprague Dawley* test rats with CAPE administration

In this study, an independent *t*-test was conducted in order to assess CAPE's effect on various biomarker expressions in the brain-injured rats on day 4 and day 7 (Table 2).

**Table 2:** Effects of CAPE on mRNA expression in brain-injured test rats.

Day	CAPE Administration	mRNA		p*
		Mean	SD	
0	(+)	5756.20	705.656	0.908
	(−)	5696.00	881.963	
4	(+)	8247.80	793.272	<0.001
	(−)	11933.20	277.915	
7	(+)	10099.00	531.490	<0.001
	(−)	13882.60	434.304	

*p value ≤ 0.05 is considered significant.

**Fig. 2:**
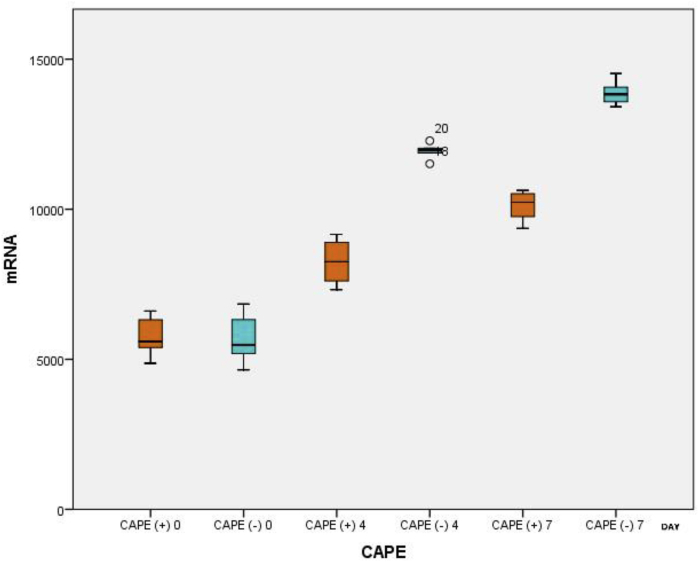
Boxplot graph of AQP-4 mRNA results in rats with and without CAPE administration.

The results in these brain-injured rats on days 4 and 7 showed a significantly lower average mRNA expression in rats treated with CAPE compared to rats that were not treated with CAPE (*p* < 0.05). As seen in Table 2, mRNA expression was higher on day seven than on day 4 (Fig. 2).

**Fig. 3:**
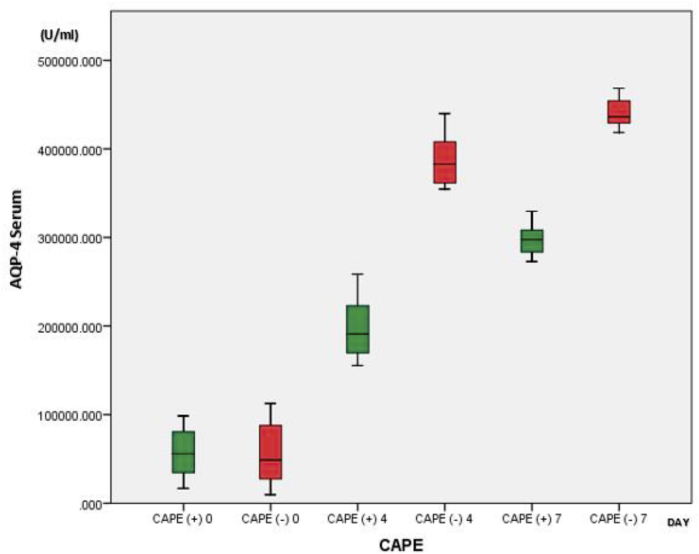
Boxplot graph of AQP-4 serum results in rats with and without CAPE administration.

The effects of CAPE on AQP4 expression in the brain-injured rats on days 4 and 7 were analyzed using the independent *t*-test. The average AQP4 expression in rats treated with CAPE was significantly lower than in rats not treated with CAPE (*p* < 0.05) (Table 3). AQP4 expression was lower on day 4 than on day 7 (Fig. 3).

**Table 3:** Effects of CAPE on AQP-4 expression in brain injured test rats.

Day	CAPE administration	AQP-4 (U/ml)		*p*
		Mean	SD	
0	(+)	57324.172	33216.815	1.00
	(−)	57324.172	42557.289	
4	(+)	199527.859	41656.8219	<0.001
	(−)	389369.781	35049.675	
7	(+)	298359.421	22115.988	<0.001
	(−)	441274.127	19889.462

**Fig. 4:**
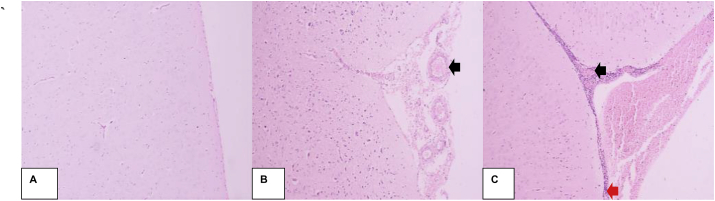
Comparison of histological features of Sprague Dawley rats' brain tissue. A) Normal brain (without brain injury): no microscopic abnormalities. B) Day 7 after trauma, CAPE (−): proliferation and dilation of blood vessels (black arrows), accompanied by hemorrhage. C) Day 7 after trauma, CAPE (+): inflammatory cell infiltrations (red arrows) and blood vessel contractions (black arrows) with hemorrhage. (HE staining, magnification 400×). (For interpretation of the references to colour in this figure legend, the reader is referred to the Web version of this article.)

Based on the trauma models, experimental analysis on brain tissue damage showed an effect of CAPE in a seventh-day result of decreased proliferation and dilation of blood vessels (Fig. 4).

## 4 Discussion

The definition of head injury is an injury to brain tissue not due to degenerative or congenital processes but instead due to external impacts that can result in a decrease or change in the status of consciousness. Head injury is the leading cause of accident-related death for people under the age of 40 years. Every year approximately 10 million people are hospitalized due to head injuries worldwide [[25], [26], [27]].

Head injury is a major cause of mechanical brain cell injury and is also a catalyst for secondary damage, which develops immediately after the primary damage occurs [1]. Secondary non-mechanical injuries are progressive and start developing in the first post-trauma hour. One secondary condition triggered by head injury is cerebral edema [28], an accumulation of intracellular and/or extracellular brain fluid [29]. This condition is characterized by swelling of the brain tissue due to a progressive increase in brain fluid levels, which can occur as a result of ischemia [30], trauma [31], tumors, and inflammation [32].

This study examined the use of CAPE in reducing the formation of cerebral edema in a head injury. This study aimed to prove whether CAPE plays a role in reducing AQP4 levels and decreasing mRNA expression of AQP4 mRNA.

The experimental animal in this study was a *Sprague Dawley* strain of *Rattus norvegicus*, with an average body weight of 290.07 (±10.48) grams; no significant difference was found in the distribution of body weight of these rat subjects. Consequently, any differences in expression of the dependent variables are expected to be the result of CAPE treatment after the induced head injury.

This study used a head injury model that modified the methods used by Marmarou [17]. To prove whether the trauma could cause brain injury, tissue histopathological examination was carried out. Observations of signs of bleeding were conducted, as well as measurements of AQP4 serum levels and AQP4 mRNA levels. CAPE was administered at a dose of 10 mg/kg, once at 30 min post-trauma and then once every day for seven days. In the results of this study, which drew on an analysis of the wound area, a noticeable decrease was seen in the group treated with CAPE, in comparison to the group not treated with CAPE.

The process of cerebral edema is mediated by several mediators, including AQP4 and cytokines. AQP4 is crucial in the development of cerebral edema [30]. Several studies have shown increased levels of AQP4 after a head injury and have demonstrated its role in the incidence of cerebral edema. Accordingly, the use of AQP4 inhibiting agents is thought to play a role in controlling cerebral edema [33,34].

In this study, AQP4 levels increased until the 7th day. With CAPE treatment, AQP4 levels were found to be lower than without CAPE treatment. This process shows that CAPE can reduce the occurrence of cerebral edema. This process is in line with the reduction in edema symptoms and the improvement in functional status associated with a return of AQP4 to normal levels. The speed of improvement of AQP4 levels after CAPE treatment indicates post-translational modification, which plays a role in protein synthesis [35].

Increased levels of AQP4 were also observed at the level of AQP4 mRNA expression. Real-time quantitative PCR found a significant increase in mRNA expression in the control group compared to the treatment group. This indicates an effectiveness of CAPE in the process of decreasing cerebral edema caused by a head injury—especially the cytotoxic process of edema characterized by the release of AQP4 by astrocytes, which undergoes a neuroinflammatory process [35].

This study examined AQP4 expression, which was observed by assessing the serum levels and the mRNA amplification. The results of post-trauma AQP4 expression after CAPE administration showed a significant decrease in serum levels.

Most brain diseases, including traumatic head injuries, present the characteristic feature of edema, which is the accumulation of fluid produced by the brain as a result of dysfunction of osmotic homeostasis. The main consequence of edema is brain swelling, which worsens other secondary injuries, such as decreased brain perfusion [36]. Edema has been a common topic in clinical and pre-clinical science for many years, but the molecular and cellular events in the formation and resolution of edema are still poorly understood. Furthermore, no efficient treatment has been developed to prevent or limit the formation of edema or expansion of brain damage. Thus, this research on CAPE's effect on the AQP4 protein in the brain is a stepping stone to developing treatment to counter the edema process.

## 5 Conclusion

Administration of CAPE in a rat subject with a head injury can reduce cerebral edema, mediated by AQP4. These findings warrant further research that observes cerebral edema in rat subjects, measured directly by computed tomography (CT) scans and anti-AQP4 immunohistochemistry expression.

## 6 Provenance and peer review

Not commissioned, externally peer reviewed.

## Ethical approval

All procedure for Animal experiment has been approved by Ethics Commission Faculty of Medicine, Hasanuddin University Number: 771/UN4.6.4.5.31/PP36/2019.

## Sources of funding

No funding or sponsorship.

## Author contribution

RAN, AAI, MH, PP, DHL, and ISM wrote the manuscript and participated in the study design. RAN, AAI, PP, DHL, ISM, HW, WS, MNM, and KIN drafted and revised the manuscript. RAN, AAI, PP, DHL, and ISM, performed head trauma treatment and surgery. RAN, AAI, MH and PP performed bioinformatics analyses and revised the manuscript. All authors read and approved the final manuscript.

## Registration of research studies

None.

## Guarantor

Rizha Anshori Nasution.

## Declaration of competing interest

The authors declare that they have no conflict of interests.
